# Flood Detection/Monitoring Using Adjustable Histogram Equalization Technique

**DOI:** 10.1155/2014/809636

**Published:** 2014-01-02

**Authors:** Fakhera Nazir, Muhammad Mohsin Riaz, Abdul Ghafoor, Fahim Arif

**Affiliations:** ^1^Department of Computer Software Engineering, College of Signals, National University of Sciences and Technology (NUST), Islamabad, Pakistan; ^2^Department of Electrical Engineering, College of Signals, National University of Sciences and Technology (NUST), Islamabad, Pakistan

## Abstract

Flood monitoring technique using adjustable histogram equalization is proposed. The technique overcomes the limitations (overenhancement, artifacts, and unnatural look) of existing technique by adjusting the contrast of images. The proposed technique takes pre- and postimages and applies different processing steps for generating flood map without user interaction. The resultant flood maps can be used for flood monitoring and detection. Simulation results show that the proposed technique provides better output quality compared to the state of the art existing technique.

## 1. Introduction

Remote sensing technology has played an important role in flood monitoring in recent years. This development (optical/aerial to radar remote sensing) provides all weather capability as compared to the optical sensors for the purpose of flood mapping. Flood mapping [1–3] is one of the techniques used for flood monitoring in which pre- and postflood images are compared to classify undated (nonflooded) and inundated (flooded) areas.

Initially flood monitoring was limited to satellite [4] and aerial images [5]. However due to the development of radar remote sensing, the issue of limited performance in bad weather conditions (like clouds, lightening, etc.) [6] is resolved. The use of Synthetic Aperture Radar (SAR) imagery has solved the problem of flood monitoring due to its all weather capability [7]. Broadly the flood mapping techniques are divided into supervised (which requires operator involvement), semisupervised, and unsupervised techniques.

Some popular flood mapping techniques are visual interpretation [8], segmentation [9], thresholding [10], texture matching [11], and analysis of dynamic and physical characteristic of region of interest [12]. Visual interpretation [8] is the commonly used supervised approach for flood mapping. Besides consistent results of visual interpretation, user involvement is not always practically feasible.

Segmentation (semisupervised) technique [9] was proposed to minimize the involvement of user. The technique generates a connectivity map using fuzzy logic by selecting a seed point from user. Still it depends upon correct identification of seed point (chosen by user). A fast ready flood map (without user involvement) and a detailed flood map based on seed growing mechanism were proposed in [13] to overcome empirical settings. However, the detailed flood map still needs user ability to locate the points for segmentation.

Thresholding uses certain thresholds for unsupervised floods monitoring [10]. The thresholds are selected on the basis of the output of three electromagnetic scattering models to generate fast ready maps. However, these thresholds do not work under complex environmental conditions (in that case users involvement is required for reliable results) [14]. Moreover, a universal threshold cannot be not justified for flood detection [15].

Texture matching is also used to identify water areas from images [11]. United and homogenous regions of water are segmented; chromatic and texture features are then compared to predefined samples of water. Main limitations are heavy computation time and overlapping texture features.

Different flood monitoring techniques are combined to generate inundation map [16]. The map represents the degree of belief for each pixel. However, reliable calibration and verification are not always possible. In [12], complex coherence map is used to perform an analysis on SAR data for flood monitoring and receding. This technique is not only confined to flood damage assessment but also other areas can be monitored (like earthquake). However, it depends upon the availability of optical images for observed events.

Recently, a chain of processing-based method [17] was proposed for better visual representation of an event. This method applied different processing chains (adaptive histogram clipping (AHC), histogram remapping (HR), and histogram equalization (HE)) to improve visualization. RGB image is then generated by combining the processed pre-, post- and difference images. The chain of events is performed to preserve the important information (in SAR image) [9]. However this process sometimes highlights extra details in the difference image which degrades the quality. Moreover the equalization process results in excessive contrast enhancement, which in turn gives the processed image an unnatural look.

To resolve these issues of [17], we propose a contrast enhancement-based technique to improve the visibility of resultant flood maps. The technique follows the three chains for processing proposed by Dellepiane and Angiati [17]. However, the chains are applied on the pre- and postimages for the generation of difference image only. A fast ready flood map is generated by combining the difference image with the equalized pre- and postimages. In contrast to Dellepiane and Angiati [17], we have used Adjustable HE (AHE) [18] with a low percentile value to improve the visibility. Results are evaluated using different data sets which show the significance of proposed technique.

## 2. Proposed Methodology

An AHE-based flood monitoring technique is proposed which is composed of three chains of processing. Let *I*_*X*(*l*,*m*)_ be pre-, *I*_*Y*(*l*,*m*)_ post-, and *I*_*Z*(*l*,*m*)_ difference images, where *l* ∈ [0,…, *L* − 1] and *m* ∈ [0,…, *M* − 1].

The first step is AHC, which is used to preserve the information content. The histograms of (pre-, post-, and difference) images are clipped/truncated at a specific percentile value (to remove the intensities which contain much less amount of information). The histograms of preimage *I*_*X*_ are clipped using a specific percentile value *q*. Let *h*_*X*_(*i*) be the histogram of image *I*_*X*_, where *i* represents the intensity levels [0–255]. The cumulative histogram *C*_*X*_(*i*) is

(1) $$\begin{array}{c} C_{X}\left(i\right)=\overset{i}{\underset{j=1}{\sum }}h_{X}\left(i\right). \end{array}$$

The clipping is performed as

(2) $$\begin{array}{c} q=\arg\left[C_{X}\left(i\right)\;|\;C_{X}\left(i\right)=q\times M\times L\right]. \end{array}$$

Dellepiane and Angiati [17] have used the same approach in which they used *q* = 0.98. However the issue in [17] is the excessive amount of details present in the final RGB map generated using clipped pre-, post- and difference image at proposed *q* percentile which finally contribute to flooding. To resolve this issue we used different *q* percentile value in the first step to generate the difference image. It is observed that at low percentile values required details are removed, whereas, at higher percentile values, unwanted details get more prominent, thus degrading the quality. Therefore, we have used *q* = 0.30 because it preserves the required intensity values which contribute to flooding.

In the second step (HR), the clipped histogram is remapped to the original intensity range using linear scaling. The histogram of image *I*_*X*_1__ (adaptive histogram clipped image *I*_*X*_) is remapped to full intensity range [0–255]. Let min(*I*_*X*_1__) represent the minimum of all intensities and let max(*I*_*X*_) represent the maximum of all intensities in the image. *I*_*X*_2__ (histogram remapped image *I*_*X*_1__) is given as [17]

(3) $$\begin{array}{c} I_{X_{2}}=\frac{I_{X_{1}}-\min\left(I_{X_{1}}\right)}{\max\left(I_{X_{1}}-\min\left(I_{X_{1}}\right)\right)}\times 255. \end{array}$$

In the third step (AHE), we use adaptive histogram equalization to enhance the image. Reference [17] uses traditional HE which sometimes overenhances the image and produces unwanted artifacts (roughness, etc.) of processed images (pre-, post- and difference). Furthermore, the processed images sometimes may not reveal all the details or merge the details which results in degradation of image quality. A contrast enhancement technique is required to maintain the smoothness and natural aspect of an image, for visually pleasing results. To achieve the proposed outcome, we use a new framework for histogram modification [18] to improve the visualization by preserving its details.

HE usually maps the input intensity levels *i* to the output level *X*_*i*_ according to

(4) $$\begin{array}{c} X_{i}=\left(N-1\right)\times C_{X(i)}, \end{array}$$

where *N* represents the total intensity levels in image and *C*_*X*(*i*)_ represents the cumulative histogram. This mapping is suitable for images with continues intensity levels where it perfectly equalizes the histogram. However, for digital images, traditional HE is not useful because of their discrete intensity levels [19].

In order to make it suitable for digital images, input histogram can be modified without compromising its contrast enhancement. The modified histogram can then be used as a mapping function for HE. The issues of HE are addressed by using the proposed Arici et al. [18] framework for histogram modification. The technique introduces specifically designed penalty terms which can be used to adjust the level of contrast enhancement.

Once the intensity range is remapped, AHE [18] is used to minimize the effects (like overenhancement, unusual artifacts, and unnatural look). The principle of AHE is to minimize the difference between modified *h*_*X*_*m*__ and current histogram *h* such that the modified histogram is also closer to the uniform histogram *h*_*X*_*u*__; that is,

(5) $$\begin{array}{c} \min||h-h_{X}||+\alpha ||h-h_{X_{u}}||, \end{array}$$

where *α* is used to adjust the contribution of current and uniform histogram. The modified histogram by solving (5) [18] is

(6) $$\begin{array}{c} h_{X_{m}}=\left(\frac{1}{1+\alpha }\right)\times h_{X}+\left(\frac{\alpha }{1+\alpha }\right)\times h_{X_{u}}. \end{array}$$

The modified histogram *h*_*X*_*m*__ is used to produce images *I*_*X*_3__ and *I*_*Y*_3__.

Note that conventional histogram used in [17] produces unwanted artifacts, overenhancement, and unnatural look. This is due to the fact that the goal of traditional HE is to match the input histogram with uniform distribution. However, AHE also minimizes the difference between modified and input histogram (along with the input and uniform histogram). Hence, AHE produces more reliable results for flood monitoring.

Difference image *I*_*Z*_ is then generated using *I*_*X*_3__ and *I*_*Y*_3__ [17]:

(7) $$\begin{array}{c} I_{Z}\left(l,m\right)=128+\frac{I_{X_{3}}\left(l,m\right)-I_{Y_{3}}\left(l,m\right)}{2}. \end{array}$$

Fast ready flood map is generated finally by combining adaptive histogram equalized pre- and postimages with the difference image. In [17] RGB map is generated by applying all chains of processing on pre- and postimages which are then combined with difference image. However, the processing of all images through same chains does not preserve intensity values in pre- and postimages. Hence, in our case, *I*_*X*_ and *I*_*Y*_ are only passed through the third chain of processing (AHE) to produce ${\hat{I}}_{X}$ and ${\hat{I}}_{Y}$, respectively.

Finally $I_{Z},\;{\hat{I}}_{X}$, and ${\hat{I}}_{Y}$ are combined to generate fast ready map by assigning blue, green, and red bands to pre-, post-, and difference images, respectively. The level of red color is high for pixels whose prevalue dominates and vice versa. In RGB image, medium to dark red color represents permanent water like rivers and dark blue color represents the flooded areas.

The reason for using only third step AHE for RGB generation is to preserve intensity values of pre- and postimages that maintain the details. The purpose of using processed pre- and postflooded images for difference image generation is to remove the intensities which contribute very low in flooded areas.

**Figure 1:**
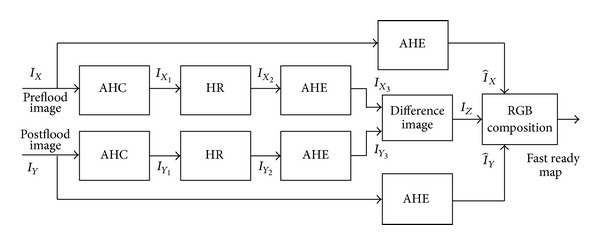
Flow chart of the proposed algorithm.

Figure 1 shows the block diagram of proposed technique.

## 3. Simulation and Results

**Figure 2:**
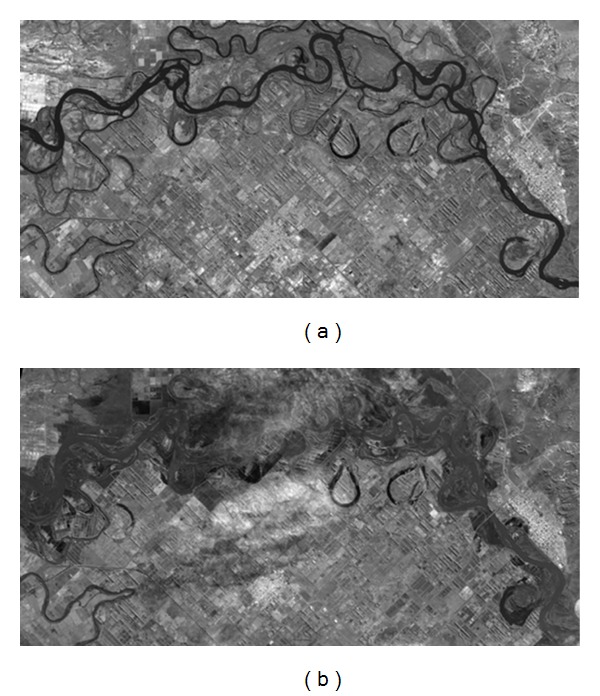
Original images of Choele Choel City, Argentina, observed by “Daichi” (ALOS). (a) Preflooded image acquired on April 29, 2006. (b) Postflooded image acquired on July 30, 2006.

For evaluation of existing and proposed techniques, flood-occurring areas in Choele Choel City, Argentina, are considered. The images are observed by “Daichi,” Advance land observing satellite on April 29 (preflooded image, shown in Figure 2(a)), and July 30, 2006 (postflooded image, shown in Figure 2(b)), respectively. Second data set includes the images of Tomakomai, Japan, acquired by Phased Array Type L-band SAR (PALSAR) using H/V polarization on August 19, 2006, in Figure 6(a) and V/V polarization on August 19, 2006, in Figure 6(b).

**Figure 3:**
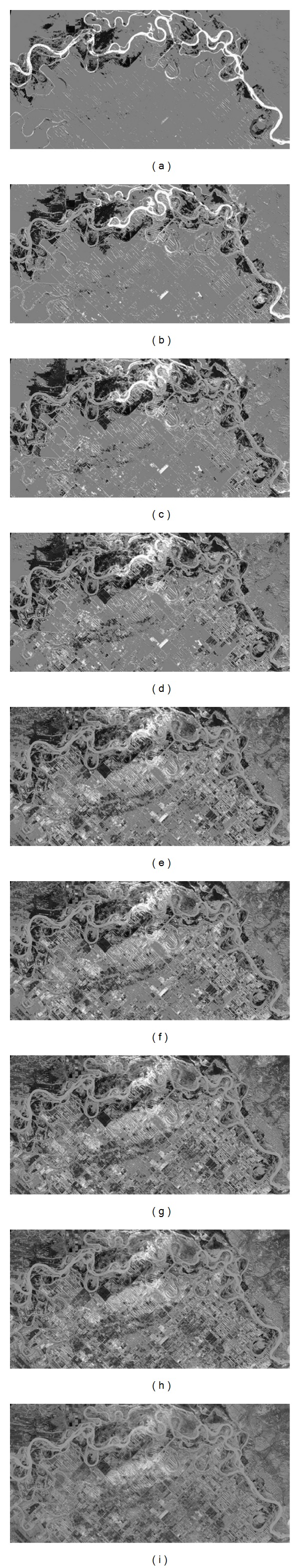
Difference images *I*_*Z*_ for different percentiles values: (a) at *q* = 0.1, (b) at *q* = 0.2, (c) at *q* = 0.3, (d) at *q* = 0.4, (e) at *q* = 0.5, (f) at *q* = 0.6, (g) at *q* = 0.7, (h) at *q* = 0.8, and (i) at *q* = 0.98.

Figure 3 shows the variation in the difference image with respect to percentile value *q*. By increasing *q*, the details in the image increase (and vice versa). In Figure 3(a) we can notice that the ground area around the river is dim, which becomes quite visible in Figure 3(b) but the flood water is not so clear. At percentile value *q* = 0.30 (in Figure 3(c)), the ground area, permanent water, and flood are visible to the required level. As we move to higher percentile values (*q* > 0.3) ground area becomes more prominent gradually which contributes to the change area in final RGB composition. This effect can be observed in Figures 3(d)–3(i).

**Figure 4:**
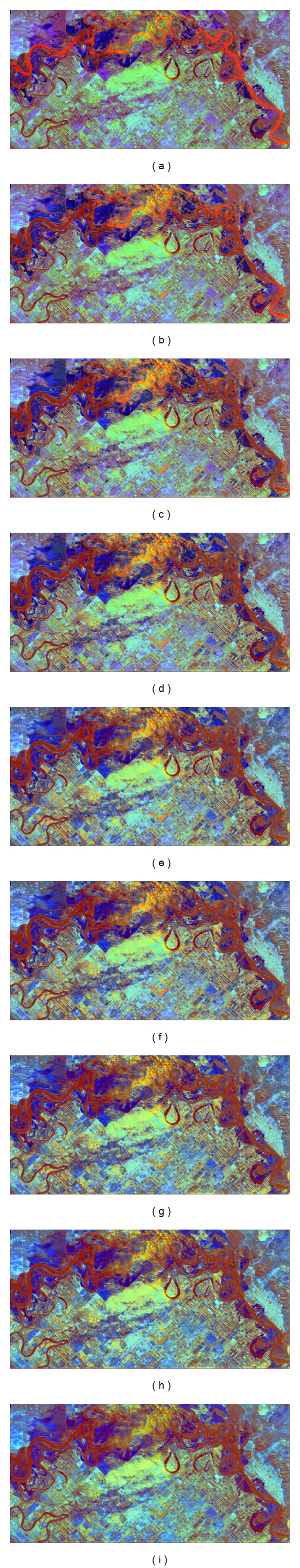
RGB images for different *q* percentiles values: (a) at *q* = 0.1, (b) at *q* = 0.2, (c) at *q* = 0.3, (d) at *q* = 0.4, (e) at *q* = 0.5, (f) at *q* = 0.6, (g) at *q* = 0.7, (h) at *q* = 0.8, and (i) at *q* = 0.98.

Figure 4 represents RGB images, generated by respective difference images (given in Figure 3). In Figures 4(a) and 4(b) the flooded area is dim, which fades away around the river. In Figure 4(c) (at *q* = 0.30), the flooded area around the river (at the top center of image) becomes quite visible to the acceptable level. The light ink blue area (at the bottom center of image) is reflecting the flooded pixels at the required level. For higher percentile values (*q* > 0.3) in Figures 4(d)–4(g), RGB images gradually increase the flooded areas at the bottom center of image (in dark ink blue color). The visibility of flooded areas on the top center of image is also not good. The results are quite obvious in Figures 4(h) and 4(i), where flooded areas are more faded around the river, but a lot of flooded areas are seen at the bottom center.

**Figure 5:**
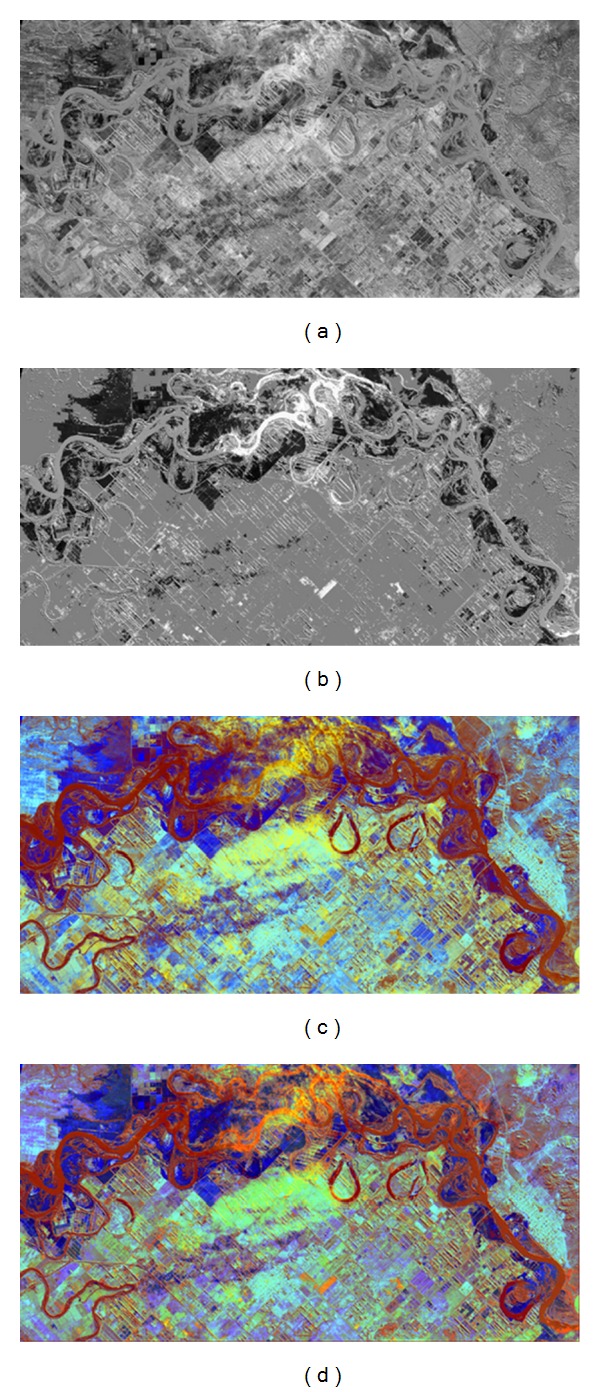
Evaluation of results using images of flood that occurred in Choele Choel City, Argentina. (a) Difference image obtained by Dellepiane and Angiati [17] approach. (b) Difference image obtained by proposed technique. (c) Fast ready map generated using Dellepiane and Angiati [17] approach. (d) Fast ready map generated using proposed technique.

Figure 5 provides comparison of the proposed technique and Dellepiane and Angiati [17] technique.  Figure 5(a) is a difference image generated using Dellepiane and Angiati [17] technique and Figure 5(b) is generated using the proposed methodology. There is a clear difference in details in these images.  Figure 5(a) shows the ground details more prominently while Figure 5(b) highlights the major required details comparatively. These differences in details contribute a lot to their respective RGB (Figures 5(c) and 5(d)). We can notice the flooded area (in Figure 5(c)) around river (at the top center) is blur (not clear) which degrades visibility. A very high contribution of irrelevant details of difference image in RGB is visible (the blue color at the bottom center and dark blue color at the top right corner of image).  Figure 5(d) shows better visibility of flooded area around river (at the top center), low blue color (at the top right corner), and low flooded areas (at the bottom center of image). One can clearly notice the difference in contrast/details of ground area and the contrast of river with flooded areas.

**Figure 6:**
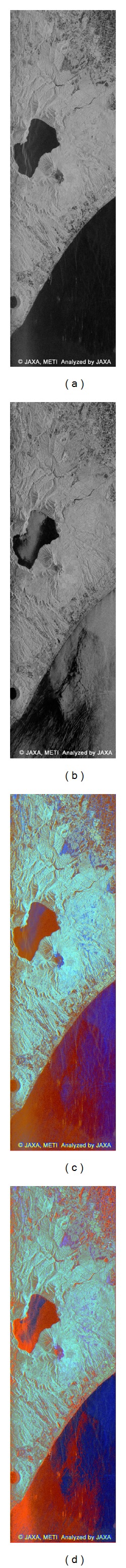
Evaluation of results using images of Tomakomai, Japan. (a) Preimage acquired on 19 August 2006. (b) Postimage acquired on 19 August 2006. (c) Fast ready map generated using Dellepiane and Angiati [17] approach. (d) Fast ready map generated using proposed technique.

Figure 6(c) is the RGB map generated using Dellepiane and Angiati [17] technique. Although the image (in Figure 6(c)) is enhanced, it highlights the irrelevant details which contribute to flooding (see the blue colored areas at the right center of image). The details at the top right flooded area (in Figure 6(d)) are clear as compared to the flooded areas in Figure 6(c). Figure 6(c) mixes up the details due to overenhancement at the areas around the river while these areas are more clear in Figure 6(d) (in red colors). Figure 6(c) produces unnatural ground details; however more smoothness of image is seen in Figure 6(d) that preserves the natural effect of image to some extent.

## 4. Conclusion

A contrast enhancement-based flood mapping approach for SAR images is proposed which is composed of three steps (histogram adaptive clipping, remapping, and adjustable histogram equalization). Pre- and postflooded images are processed using different processing chains and the difference image is produced (by pre- and postimages). A fast ready flood map is then generated, using the combination of processed pre- and postimages (only the third step is applied) with difference images. A specific contrast enhancement technique AHE is used as a third step to remove the overenhancement produced by HE. The proposed technique is an improvement in existing state of the art, which suffers from unwanted details, unnatural look, and overenhancement of the image. The technique produces visually pleasing results by suppressing the irrelevant details and minimizing overenhancement, thus maintaining quality. Simulation results show the significance of proposed technique.
